# Gut Microbes and Host Physiology: What Happens When You Host Billions of Guests?

**DOI:** 10.3389/fendo.2014.00091

**Published:** 2014-06-13

**Authors:** Jennifer L. Pluznick

**Affiliations:** ^1^Department of Physiology, Johns Hopkins University School of Medicine, Baltimore, MD, USA

**Keywords:** gut microbiota, gut bacteria, host physiology, short-chain fatty acids, gut metabolites

The gut microbiota has recently emerged as an important, and previously unappreciated, player in host physiology (1). In particular, the gut microbiota contributes to a variety of physiological and pathophysiological processes in the host including immune disorders (2–4), atherosclerosis (5), irritable bowel syndrome (6, 7), blood pressure regulation (8), and chronic kidney disease (9, 10). Bacteria residing in the human gut are an important component of human physiology: the total wet weight of gut microbes in the human has been estimated to be 175 g–1.5 kg (11, 12), and the cells of the microbiota outnumber human cells by 10:1 (1). These bacteria interact with the immune system of the host (13), and secrete a variety of metabolites, which enter host circulation and can affect a variety of physiological parameters (8, 14), reviewed in Ref. (15). In fact, metabolites produced by the gut microbiota have been found to play key roles in renal disease (16), blood pressure regulation (8), and immune disorders (2–4). Therefore, just as we consider the genetic background of an animal or an individual to be an important contributing factor to their physiology, so too must we consider the genetic background of the microbiota associated with that animal.

Gut microbiota vary greatly amongst laboratory animals, and these differences result in notable differences in experimental results. Mice of the same strain from different vendors have different microbiota profiles (17), and similarly, the same mice housed at different institutions have different microbiota profiles (18, 19). Conversely, inoculating two different inbred mouse strains with the same gut bacteria leads to differences in host gene expression between the two mouse strains (20). Clearly, there is a complex interplay between the genetics of the microbiota and that of the host organism, which has only recently begun to be appreciated.

## Gut Microbiota as an Experimental Parameter

Examples in the literature have highlighted the important and unexpected ways in which gut microbiota can affect a variety of experimental parameters. In a series of studies, Vijay-Kumar et al. (13, 21) reported that although TLR5 null animals initially had a colitis phenotype, when these mice were “rederived” and their gut microbiota altered, the colitis phenotype was greatly attenuated, and instead the null animals exhibited metabolic syndrome. In addition, Lathrop et al. put forward a model by which T-cells are educated not only by self/non-self mechanisms, but also by microbiota-derived “non-self” antigens (22). Accordingly, they found that the presence or absence of microbiota determined whether T cells would induce colitis in mice. Finally, Yang et al. reported that when the same knockout mice were housed at two different institutions, they had markedly different microbiota profiles – and the mice at one institution (MIT) were quite susceptible to colitis, whereas mice at the other institution (MHH) failed to develop any significant pathology under the same conditions (19). Unequivocally, altering gut microbiota – even by housing animals at different institutions – can have dramatic effects on the phenotype observed.

## Gut Microbiota and Obesity and Diabetes

It is important to note that not only can microbiota affect host physiology, but the gut microbiota are not necessarily stable over time. Rather, gut microbiota can change or shift as a result of experimental manipulation (in animals) or changes in lifestyle or nutrition (in humans). It is now appreciated that there are “shifts” in microbiota that occur in obesity in mice, rats, and humans (23–26). In one study, Turnbaugh et al. (25) examined human female twin pairs concordant for leanness or obesity, and found that obesity was associated with phylum-level changes in microbiota. In this study, both monozygotic and dizygotic female twin pairs (and their mothers, where available) were analyzed. Analysis of fecal samples revealed that obese individuals have reduced gut microbiota diversity, and tended to have less Bacteroidetes and more Actinobacteria. The authors suggest that conditions of “abnormal energy input” (i.e., obesity) may favor the growth of a reduced diversity community. In support of these findings, a separate study reported that ob/ob mice had a reduction in Bacteroidetes (27) as compared to ob/+ or wild-type siblings. Furthermore, in 12 obese humans who lost weight over 1 year by consuming either a fat restricted or caloric restricted diet, there was a relative increase in the abundance of Bacteroidetes over time (28). Impressively, the increase in Bacteroidetes in these individuals correlated with weight loss. Indeed, there is an increasingly convincing link between gut microbiota and obesity. These findings highlight the importance of considering and documenting gut microbiota composition in studies of obesity, as a change in gut microbiota structure has a clear tie to host pathology.

In addition, it has also been demonstrated that type 2 diabetes in humans is associated with changes in the gut microbiota (not surprising, given the correlation between obesity and type 2 diabetes) (29–33). Specifically, it has been reported that butyrate-producing bacteria are reduced in type 2 diabetes (32), that Bifidobacterium is lowered (33), and that Firmicutes is decreased (30). Intriguingly, it was also reported that the ratio of Bacteroidetes to Firmicutes (as well as the ratio of Bacteroides–Prevotella to *C. Coccoides*–*E. rectale*) correlated with plasma glucose levels. Subsequently, it has been suggested that manipulating gut microbiota may be a therapeutic opinion for obesity and/or type 2 diabetes (34). As reviewed in depth by Kootte et al. (34), in addition to exploring possible roles for prebiotics, probiotics, and antibiotics as potential therapies, we must also better understand the changes in microbiota which appear to accompany known treatments for obesity (i.e., bariatric surgery). In addition, we must continue to explore a potential role for a variety of microbiota metabolites in order to better understand how and why changes in microbiota affect the physiology of the host.

## What’s a Scientist to Do?

Knowing that gut microbiota play such an important and dynamic role in host physiology, going forward we should take into account (or at least, document) the gut microbiota present in whole-animal physiology experiments. This is especially important in research focused on obesity and diabetes, as these are areas in which gut microbiota changes have been associated with host pathology. As the study of the gut microbiota requires a specialized and complex set of knowledge, it may be prudent for Universities, Companies, or other Research Entities to establish core facilities and/or collaborations to help facilitate such measurements. In addition, when differences are found in measured physiological parameters between researchers at different institutions, gut microbiota should be considered as one potential explanation. Finally, as different strains of microbiota produce different metabolites, metabolomics may also be a useful tool to help us understand the “end effect” of microbiota on host function.

## Conclusion

The gut microbiota is a complex tangle of organisms, which easily outnumber the number of cells in the host. When considering processes in the context of whole-animal physiology, we must also consider the contribution of these microorganisms and their metabolites. The literature is rife with examples of phenotypes which were not easily replicated by other groups – even when using the “same mice” – and we should consider whether some of these examples may be, in fact, due to the influence of gut microbiota. In the future, it would be ideal for researchers to report at least a basic characterization of gut microbiota in research animals so that any “institution-specific” effects can later be examined. Understanding not only the host and the microbiota, but the host–microbiota interactions, will ultimately give us a richer and fuller understanding of host physiology.

## Conflict of Interest Statement

The author declares that the research was conducted in the absence of any commercial or financial relationships that could be construed as a potential conflict of interest.
